# Proposing a novel molecular subtyping scheme for predicting distant recurrence-free survival in breast cancer post-neoadjuvant chemotherapy with close correlation to metabolism and senescence

**DOI:** 10.3389/fendo.2023.1265520

**Published:** 2023-10-12

**Authors:** Jin Huang, Jian-Lin Zhang, Lin Ang, Ming-Cong Li, Min Zhao, Yao Wang, Qiang Wu

**Affiliations:** ^1^ Department of Pathology, The Second Affiliated Hospital of Anhui Medical University, Hefei, Anhui, China; ^2^ Department of Pathology, The First Affiliated Hospital of Anhui Medical University, Hefei, Anhui, China; ^3^ Department of Emergency Surgery, The First Affiliated Hospital of Anhui Medical University, Hefei, Anhui, China; ^4^ Department of Pathology, The Second People’s Hospital of Hefei, Hefei Hospital Affiliated to Anhui Medical University, Hefei, Anhui, China; ^5^ Digestive Endoscopy Department, Jiangsu Provincial People’s Hospital, The First Afliated Hospital of Nanjing Medical University, Nanjing, China

**Keywords:** breast cancer, bioinformatics analysis, biomarker, neoadjuvant therapy, machine learning

## Abstract

**Background:**

High relapse rates remain a clinical challenge in the management of breast cancer (BC), with distant recurrence being a major driver of patient deterioration. To optimize the surveillance regimen for distant recurrence after neoadjuvant chemotherapy (NAC), we conducted a comprehensive analysis using bioinformatics and machine learning approaches.

**Materials and methods:**

Microarray data were retrieved from the GEO database, and differential expression analysis was performed with the R package ‘Limma’. We used the Metascape tool for enrichment analyses, and ‘WGCNA’ was utilized to establish co-expression networks, selecting the soft threshold power with the ‘pickSoftThreshold’ algorithm. We integrated ten machine learning algorithms and 101 algorithm combinations to identify key genes associated with distant recurrence in BC. Unsupervised clustering was performed with the R package ‘ConsensusCluster Plus’. To further screen the key gene signature of residual cancer burden (RCB), multiple knockdown studies were analyzed with the Genetic Perturbation Similarity Analysis (GPSA) database. Single-cell RNA sequencing (scRNA-seq) analysis was conducted through the Tumour Immune Single-cell Hub (TISCH) database, and the XSum algorithm was used to screen candidate small molecule drugs based on the Connectivity Map (CMAP) database. Molecular docking processes were conducted using Schrodinger software. GMT files containing gene sets associated with metabolism and senescence were obtained from GSEA MutSigDB database. The GSVA score for each gene set across diverse samples was computed using the ssGSEA function implemented in the GSVA package.

**Results:**

Our analysis, which combined Limma, WGCNA, and machine learning approaches, identified 16 RCB-relevant gene signatures influencing distant recurrence-free survival (DRFS) in BC patients following NAC. We then screened GATA3 as the key gene signature of high RCB index using GPSA analysis. A novel molecular subtyping scheme was developed to divide patients into two clusters (C1 and C2) with different distant recurrence risks. This molecular subtyping scheme was found to be closely associated with tumor metabolism and cellular senescence. Patients in cluster C2 had a poorer DRFS than those in cluster C1 (HR: 4.04; 95% CI: 2.60–6.29; log-rank test p < 0.0001). High GATA3 expression, high levels of resting mast cell infiltration, and a high proportion of estrogen receptor (ER)-positive patients contributed to better DRFS in cluster C1. We established a nomogram based on the N stage, RCB class, and molecular subtyping. The ROC curve for 5-year DRFS showed excellent predictive value (AUC=0.91, 95% CI: 0.95–0.86), with a C-index of 0.85 (95% CI: 0.81–0.90). Entinostat was identified as a potential small molecule compound to reverse high RCB after NAC. We also provided a comprehensive review of the EDCs exposures that potentially impact the effectiveness of NAC among BC patients.

**Conclusion:**

This study established a molecular classification scheme associated with tumor metabolism and cancer cell senescence to predict RCB and DRFS in BC patients after NAC. Furthermore, GATA3 was identified and validated as a key gene associated with BC recurrence.

## Introduction

Breast cancer (BC) is a leading cause of cancer among women worldwide, with a constant rise in global morbidity rates (1). Annually, approximately 1.7 million new cases of BC and 0.5 million BC-related deaths are reported (2). Neoadjuvant chemotherapy (NAC) is a treatment strategy that involves administering chemotherapy to treat invasive BC before local treatment (3). NAC has been shown to decrease preoperative tumor volume, facilitating complete resection of BC. Furthermore, it is also used to estimate chemo-sensitivity and eliminate occult metastasis (4). Therefore, NAC has become a standard treatment for advanced BC, especially using anthracycline followed by taxane (5). However, high rates of relapse following treatment remain a significant challenge in BC management (6). Recurrence, including local/regional cancer recurrence, distant recurrence/metastasis, and contralateral primary breast cancer (7), is a major cause of death among BC patients. While locoregional recurrences represent significant clinical challenges, distant recurrence remains the leading cause of deterioration in BC patients.

In light of the high degree of heterogeneity in BC, adopting a predictive, preventive, and personalized medicine approach represents a promising strategy for optimizing treatment outcomes and enhancing patient prognoses (8). A critical element of successful clinical management involves incorporating reliable molecular biomarkers, encompassing both early diagnostic and prognostic biomarkers to identify patients requiring prompt and aggressive management, as well as predictive biomarkers to forecast and stratify responses to novel targeted therapies (8). Recently, microarray technologies have demonstrated immense potential for high-throughput studies of gene expression, uncovering the molecular mechanisms of tumor occurrence, development, metastasis, and recurrence (9). Bioinformatics analysis has the capability to reveal heterogeneity within molecular subtypes of various cancers (10–12). While several studies have explored biomarkers for BC distant recurrence, including residual cancer burden (RCB) (13), few have delved into the pre-NAC risk of post-NAC distant recurrence through bioinformatics analysis. In this study, we collected microarray data from pre-NAC BC patients and developed a novel molecular subtyping scheme to identify the risk of distant recurrence after NAC, thereby contributing to the enhancement of personalized clinical management and treatment regimens for BC.

## Materials and methods

### Microarray datasets acquisition

We conducted a comprehensive search of the Gene Expression Omnibus (GEO) database to identify and evaluate microarray datasets (14). Our inclusion criteria for the microarray datasets were as follows: (1) the samples were collected from patients who received neoadjuvant taxane-anthracycline chemotherapy; (2) the samples were obtained prior to the initiation of NAC; (3) the follow-up period included the RCB index. We identified two GEO datasets that met these inclusion criteria (GSE25066 and GSE32603). The microarray data and clinical information for GSE25066 (n=508) and GSE32603 (n=248) were obtained from the GEO database (https://www.ncbi.nlm.nih.gov/geo). We utilized the GSE25066 cohort, which comprised a relatively large number of samples, as the training dataset, while GSE32603 served as the validation dataset. Clinical data for BC patients in the GSE25066 cohort are presented in **Table 1**.

**Table 1:** Clinical data of BC patients from GSE25066 cohort.

Characteristics	Total (N=508)
RCB class	
0/I	118 (23.23%)
II/III	299 (58.86%)
Unknown	91 (17.91%)
AGE (years)	
Mean ± SD	49.80 ± 10.46
Median[min-max]	49.00[24.00,75.00]
Grade	
1	32 (6.30%)
2	180 (35.43%)
3	259 (50.98%)
Indeterminate	15 (2.95%)
Unknown	22 (4.33%)
GGI class	
High	336 (66.14%)
Low	172 (33.86%)
Pathologic Response	
RD	389 (76.57%)
pCR	99 (19.49%)
Unknown	20 (3.94%)
ER status	
Negative	134 (43.23%)
positive	176 (56.77%)
PR status	
Negative	258 (50.79%)
Indeterminate	5 (0.98%)
positive	243 (47.83%)
Unknown	2 (0.39%)
Her2 status	
Negative	485 (95.47%)
Indeterminate	4 (0.79%)
positive	6 (1.18%)
Unknown	13 (2.56%)
Pam50 class	
Basal	189 (37.20%)
Her2	37 (7.28%)
LumA	160 (31.50%)
LumB	78 (15.35%)
Normal	44 (8.66%)
T stage	
T0	3 (0.59%)
T1	30 (5.91%)
T2	255 (50.20%)
T3	145 (28.54%)
T4	75 (14.76%)
AJCC stage	
I	8 (1.57%)
IIA	121 (23.82%)
IIB	151 (29.72%)
IIIA	121 (23.82%)
IIIB	80 (15.75%)
IIIC	23 (4.53%)
Inflammatory	4 (0.79%)
Clinical nodal status	
N0	157 (30.91%)
N1	244 (48.03%)
N2	66 (12.99%)
N3	41 (8.07%)

### Gene differential analysis and enrichment analysis

We conducted differential expression analysis using the R package ‘Limma’ (15). Differentially expressed genes (DEGs) were identified with the following criteria: |Log2FC| > 1 and FDR < 0.05. For DEG identification between different RCB index groups (RCBII/III vs. RCB0/I), we performed Limma analysis. Enrichment analyses were carried out using the Metascape tool (16), with all parameters set to the recommended defaults. Enrichment terms meeting the criteria of p-value < 0.01, minimum count of 3, and enrichment factor > 1.5 were selected for further analysis.

The Metascape tool employed hierarchical clustering to categorize enrichment terms into distinct clusters based on screening criteria of kappa scores = 4 and similarity > 0.3. From each cluster, we selected the representative term with the minimum p-value. Our findings are summarized through a presentation of the top 20 clusters, each accompanied by their enriched terms.

We obtained GMT files containing gene sets linked to metabolism and senescence from the GSEA MutSigDB database, following selection based on default settings. The GSVA score for each gene set across diverse samples was computed using the ssGSEA function, which is implemented in the GSVA package. The GSVA scores for gene sets associated with metabolism and senescence were utilized to represent the metabolic and senescent states in the studied samples.

### Weighted correlation network analysis

We utilized microarray data from samples with RCB index information in the GSE25066 cohort as input files for the R package ‘WGCNA’ to establish co-expression networks (17). WGCNA was executed with the default-recommended parameters, with the parameter settings of a minimum module size of 30 and a merging threshold of 0.25. For the establishment of co-expression networks, a soft threshold power was implemented to distinguish modules exhibiting distinct expression patterns. The selection of the soft threshold power was performed using the ‘pickSoftThreshold’ algorithm from the WGCNA R package (18). We conducted Pearson’s correlation analysis to estimate the correlation between Module Eigengenes (MEs) and the RCB index. The module with the highest Pearson’s coefficient was then identified as the key module most relevant to the RCB index.

### Machine learning framework

In order to identify key genes associated with distant recurrence in breast cancer, a machine learning framework was utilized that integrated ten different machine learning algorithms and 101 algorithm combinations. The employed machine learning algorithms encompassed a range of models, including random survival forest (RSF), elastic network (Enet), Lasso, Ridge, stepwise Cox, CoxBoost, partial least squares regression for Cox (plsRcox), supervised principal components (SuperPC), generalised boosted regression modelling (GBM), and survival support vector machine (survival-SVM). The methodology comprised the use of an input file derived from the intersection of Limma-DEGs and WGCNA-key module, performing 101 algorithmic combinations on the input file to develop prediction models via leave-one-out cross-validation (LOOCV) in the GSE25066 cohort, validating all models in various AJCC stages, assessing the Harrell’s concordance indexes (C-index) for all models across the complete GSE25066 cohort and different AJCC stages, and selecting the model with the highest average C-index as the optimal model.

### Consensus clustering

Unsupervised clustering was conducted using the ‘ConsensusCluster Plus’ R package. Agglomerative PAM clustering was performed using 1-Pearson correlation distances and resampling 80% of the samples for 10 iterations. The optimal number of clusters was determined by analyzing the empirical cumulative distribution function (CDF) plot. Microarray data’s gene expression values were used as input files for the cluster analysis.

### Genetic perturbation similarity analysis

Genetic perturbation methods, such as siRNA, shRNA, and CRISPR/Cas9, are considered indispensable in scientific research. When investigating the mechanism of a specific gene in cells, RNA-seq is commonly performed following gene knockdown or knockout. The GPSA database (http://guotosky.vip:13838/GPSA/) was utilized to identify genes that induce similar downstream effects to the input data when knocked down or knocked out. The GPSA database contains a collection of 6,096 gene sets derived from 3,048 gene perturbation RNA-seq datasets (refer to **Supplementary Table 1**). These gene sets are categorized based on upregulation or downregulation patterns. Subsequently, GSEA analysis was conducted using the complete input dataset in conjunction with the aforementioned 6,096 gene sets. The GPSA applies filters to gene set terms using the following principles: 1) gene set terms from the same datasets should both exhibit enrichment, and 2) the NES (Normalized Enrichment Score) of the two gene set terms should be opposite.

### Analysis of single-cell RNA sequencing data

The scRNA-seq expression profile matrix for GSE114727 (in-Drop) was obtained from the GEO database. The cellular annotations for GSE114727 were established using the Tumour Immune Single-cell Hub (TISCH) database (12, 19). The expression levels of individual genes were compared between different cell types using median values. UMAP analysis was performed on the expression levels of genes in the ‘Hallmark-Estrogen Response Early’ and ‘Hallmark-Estrogen Response Late’ pathways. The landscape of 22 infiltrating immune cells was assessed using the R package ‘CIBERSORT’ in microarray datasets.

### Discovery of potential drugs by computational methods

A similarity scoring algorithm known as eXtreme Sum (XSum) was utilized to identify candidate small molecule drugs from the Connectivity Map (CMAP) database (20). The DEGs between different RCB index groups (RCBII/III vs. RCB0/I) were used as input files for the XSum algorithm. Subsequently, an XSum score was calculated for each drug in the CMap database, where a lower score indicates a higher potential for therapeutic use in reversing the high RCB.

The crystal structures of proteins encoded by the hub gene were obtained from the RCSB Protein Data Bank (PDB) website (www.rcsb.org/pdb/home/home.do) (21). Additionally, the 3D structures of small molecule drugs were retrieved from PubChem (https://www.ncbi.nlm.nih.gov/pccompound). The molecular docking process involved preparing the proteins and ligands, setting up a grid, and docking the compounds, all performed using the Schrödinger software (21). The optimal pose was selected based on the docking score and the plausibility of the molecular conformation.

### Chemical-gene interaction analysis

As endocrine-disrupting chemicals (EDCs) found in the environment can mimic endogenous hormones, they may activate molecular pathways involved in the growth and development of BC. Therefore, exposure to EDCs has been linked to a poor prognosis in patients with prostate cancer. Further research is required to fully elucidate the mechanisms by which EDCs impact BC growth and prognosis. To explore the interplay between EDCs and the RCB and DRFS of BC after NAC, we conducted an analysis utilizing the meticulously curated research studies on the Comparative Toxicogenomic Database (CTD). In our analysis, we scrutinized EDCs affecting the gene expression of all key genes previously identified. Our analysis is limited to the human species only. The information about EDCs was obtained from previous literature (22).

### Real time quantitative PCR and immunohistochemistry

RNA was extracted utilizing TRIzol reagent (Ambion, USA), followed by conversion of mRNA to cDNA using PrimeScriptTM RT Master Mix (Takara, Japan). Gene transcripts were quantified through the RT-qPCR assay utilizing ChamQ SYBR qPCR Master Mix (Vazyme, China). The relative expression levels of genes were evaluated using the 2-ΔΔCT method with GAPDH as the internal reference. To measure the expression levels of GATA3 and GAPDH, GATA3’s forward primer was 5′-AAGGCAGGGAGTGTGTGAAC-3′, and reverse primer was 5′-CGGTTCTGTCCGTTCATTTT-3′; while GAPDH’s forward primer was 5′-TGTTCGTCATGGGTGTGAAC-3′ and its reverse primer was 5′-ATGGCATGGACTGTGGTCAT-3′. The experiment was repeated thrice to calculate the average. Gene expression was determined using the RT-qPCR method. The study utilized samples from 8 BC patients from The Second Affiliated Hospital of Anhui Medical University. The samples were employed for RT-qPCR. All patients involved in the study provided informed consent prior to their inclusion in the study.

The data pertaining to IHC analysis was sourced from the HPA database. The Average Optical Density (AOD) was employed as a scoring method for statistical analysis. The professional pathologists used the ImageJ software to measure the AOD, with at least three measurements per sample taken to determine the mean AOD values.

### Meta analysis

As of July 2023, we conducted a search for BC microarray datasets in the GEO database. The included datasets fulfilled the following criteria: (1) presence of complete GATA3 gene expression data; (2) availability of RFS data in clinical information; (3) samples size not less than 10. Meta-analysis was conducted using the “meta” R package to integrate hazard ratio (HR) and 95% confidence interval (CI) data from all included cohorts. Heterogeneity between the study results was determined by the I^2^ statistics. If a significant level of heterogeneity was observed, the fixed-effect model was utilized (I^2^ < 50%, P > 0.10, Mantel-Haenszel method); alternatively, the random effect model was used (I^2^ ≥ 50%, P ≤ 0.10, Der Simonian and Laird method). Publication bias was assessed using funnel plots, and their asymmetry was measured by Begg’s test and Egger’s test.

### Data sources of mendelian randomization analysis

We conducted Mendelian randomization using summary-level data from the IEU Open GWAS database (https://gwas.mrcieu.ac.uk). Notably, all participants included in the IEU Open GWAS database provided informed consent in the corresponding original studies. The GATA3 GWAS summary dataset (GWAS ID: eqtl-a-ENSG00000107485) comprised a total of 31,684 individuals of European ancestry. The BC GWAS meta dataset (GWAS ID: ieu-a-1126) provided data on 228,951 individuals of European descent, consisting of 122,977 cases and 105,974 controls. Additionally, the ER+ BC GWAS meta dataset (GWAS ID: ieu-a-1127) included a total of 175,475 individuals of European descent, with 69,501 cases and 105,974 controls. Similarly, the ER- BC GWAS meta dataset (GWAS ID: ieu-a-1128) provided information on 127,442 individuals of European descent, with 21,468 cases and 105,974 controls. Finally, the BC Survival GWAS dataset (GWAS ID: ieu-a-1165) had a sample size of 37,954 individuals of European descent, including 2,900 individuals who had succumbed to BC.

### Instrumental variable selection

Genetic variations are employed as instrumental variables (IVs) in MR to obtain unconfounded estimates for the causal effect of an exposure of interest on an outcome variable. Initially, we identified single-nucleotide polymorphisms (SNPs) that were statistically significant (p < 5*10^-8) across the genome and were associated with the exposure. To exclude SNPs that were in significant linkage disequilibrium (LD), we adopted a clumping technique with a window size of 10,000 kb and an R^2 value < 0.001. Subsequently, we consulted the Phenoscanner database (http://www.phenoscanner.medschl.cam.ac.uk/) to explore potential associations of SNPs with confounding variables and outcomes (p < 5*10^-8), and manually removed them to ensure the independence of our genetic instrumental variables from outcomes and confounding factors. Additionally, we employed the MR-Pleiotropy Residual Sum and Outlier technique (MR-PRESSO) to identify outlier variations and account for horizontal pleiotropy in our results. Furthermore, we used the following equation to cumulatively determine the F statistics for SNPs: F = (N - k - 1)R^2/k(1 - R^2), where R^2 represents the variation in the exposure explained by each IV. The F-statistics were used to assess the strength of the instruments, with an F value greater than 10 indicating substantial statistical power.

### MR analysis

Mendelian Randomization Analyses Were Executed Using R Version 4.2.1 and the ‘TwoSampleMR’ Packages to Validate the Causal Association between Exposure and Outcome. Multiple MR approaches, including the inverse variance weighted (IVW), the weighted median (WM), the Mendelian randomization-Egger (MR-Egger) methods, simple mode, and weighted model, were employed in our investigation to ascertain the causal relationship between exposure and outcome. The IVW method was predominantly selected, as it demonstrated superior statistical validity among the available methods and consistently estimated the causal effect of exposure on the outcome.

### Statistical analyses

Statistical analyses were performed using R software (version 4.0.4). The Wilcoxon/Kruskal-Wallis Test was used to compare continuous variables, while the Chi-Square test was used to assess differences in proportion. A p-value of less than 0.05 was considered statistically significant. Subgroup comparisons were analyzed using Kaplan-Meier (KM) survival analysis for DRFS and recurrence-free survival (RFS), followed by the log-rank test. Receiver operating characteristic (ROC) curve was utilized to observe the diagnostic value. Spearman’s correlation was employed for correlation analysis.

## Results

### Identification of DEGs between RCBII/III and RCB0/I

**Figure 1:**
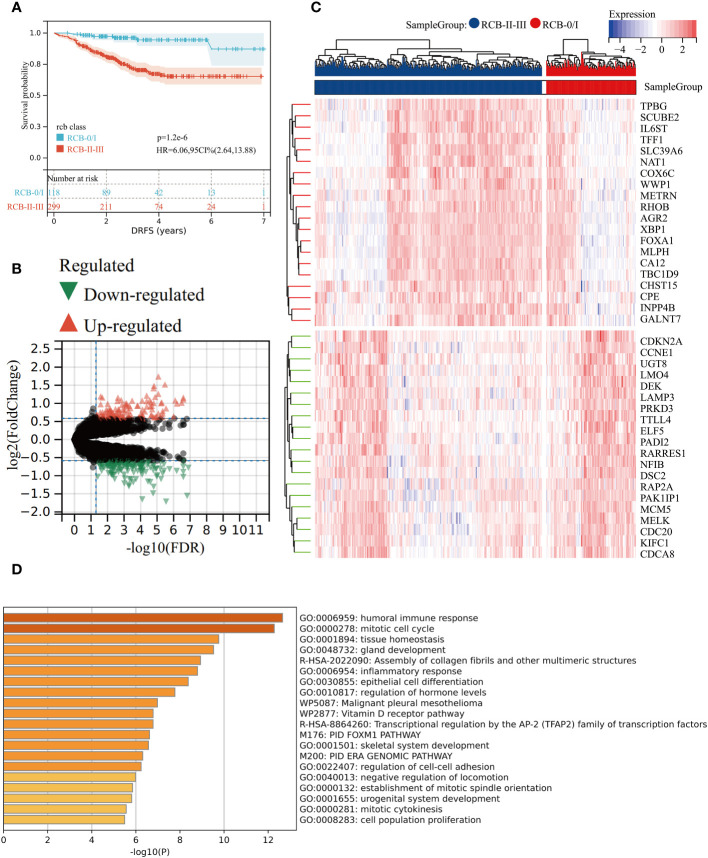
Gene differential analysis between RCB0/I and RCBII/III groups. **(A)** KM survival analysis of RCB0/I and RCBII/III groups in training dataset (GSE25066). **(B)** Volcano plot of DEGs between RCB0/I and RCBII/III groups (Green: down-regulated genes; Red: up-regulated genes). **(C)** Heatmap of the top 20 up-regulated genes and top 20 down-regulated genes according to *p* value. **(D)** The top 20 enrichment results for the DEGs based on the Metascape database.

The KM survival analysis revealed that DRFS was worse in BC patients with RCBII/III (HR: 6.06; 95% confidence interval: 2.64–13.88; log-rank test p < 0.0001; **Figure 1A**). By utilizing the “Limma” R package, we identified 181 down-regulated and 130 up-regulated genes in patients with RCBII/III before the commencement of NAC (**Figure 1B**). The heatmap displaying the top 20 up-regulated and down-regulated genes can be observed in **Figure 1C**. We subjected the 311 DEGs to analysis using Metascape tools, revealing that the top 20 enriched pathways were primarily related to humoral immune response, mitotic cell cycle, and tissue homeostasis (**Figure 1D** and **Supplementary Table 2**).

### Identification of key gene module associated with RCB index using WGCNA

**Figure 2:**
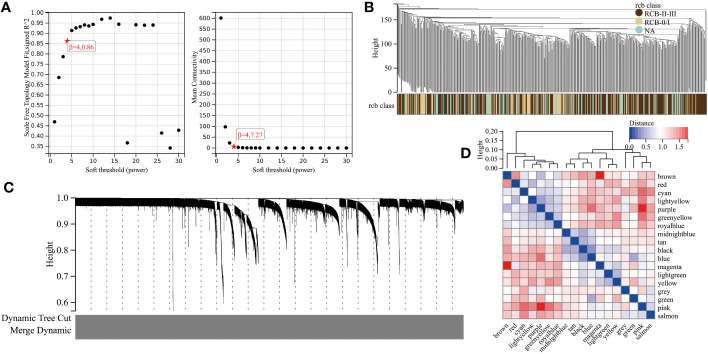
The results of WGCNA. **(A)** Determination of the soft threshold in the WGCNA algorithm. **(B)** Sample dendrogram and clinical-traits heatmap. **(C)** Cluster dendrogram and module assignment for modules from WGCNA. **(D)** Interactions among different gene coexpression modules.

**Figure 3:**
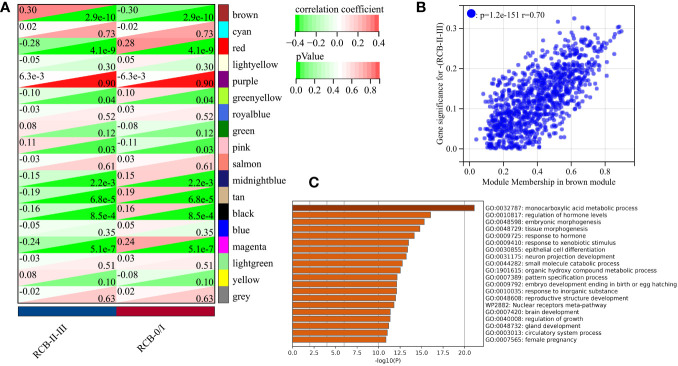
Screening results of gene modules. **(A)** Correlation between modules and clinical traits (RCB). **(B)** The scatter plot of gene significance (GS) versus module membership (MM) in the brown module. **(C)** The top 20 enrichment results for the brown module based on the Metascape database.

We utilized the “WGCNA” R package to construct a gene co-expression network with an optimal power value (β=4) (**Figure 2A**). Within the GSE25066 cohort, 6759 genes were categorized into 18 co-expression modules, inclusive of the grey module. The cluster analysis outcomes for all samples are depicted in **Figure 2B**, while the co-expression modules are visually represented using distinct colors in **Figure 2C**. The gene assignments to various modules are detailed in **Supplementary Table 3**. Furthermore, the network heatmap indicated minimal correlation among all 18 co-expression modules (**Figure 2D**). The findings from the modules correlation analysis demonstrated the brown module as the most relevant module with RCBII/III (correlation coefficient r = 0.30, p < 0.0001; **Figure 3A**). Moreover, a noteworthy positive correlation existed between the module membership (MM) of the brown module and the gene significance (GS) for RCBII/III (correlation coefficient R = 0.70, p < 0.0001; **Figure 3B**). Metascape analysis unveiled that the 1014 genes within the brown module primarily participated in monocarboxylic acid metabolic processes, regulation of hormone levels, and embryonic morphogenesis (**Supplementary Table 4**). The top 20 enriched pathways are illustrated in **Figure 3C**.

### Identification of candidate genes related to DRFS of BC

**Figure 4:**
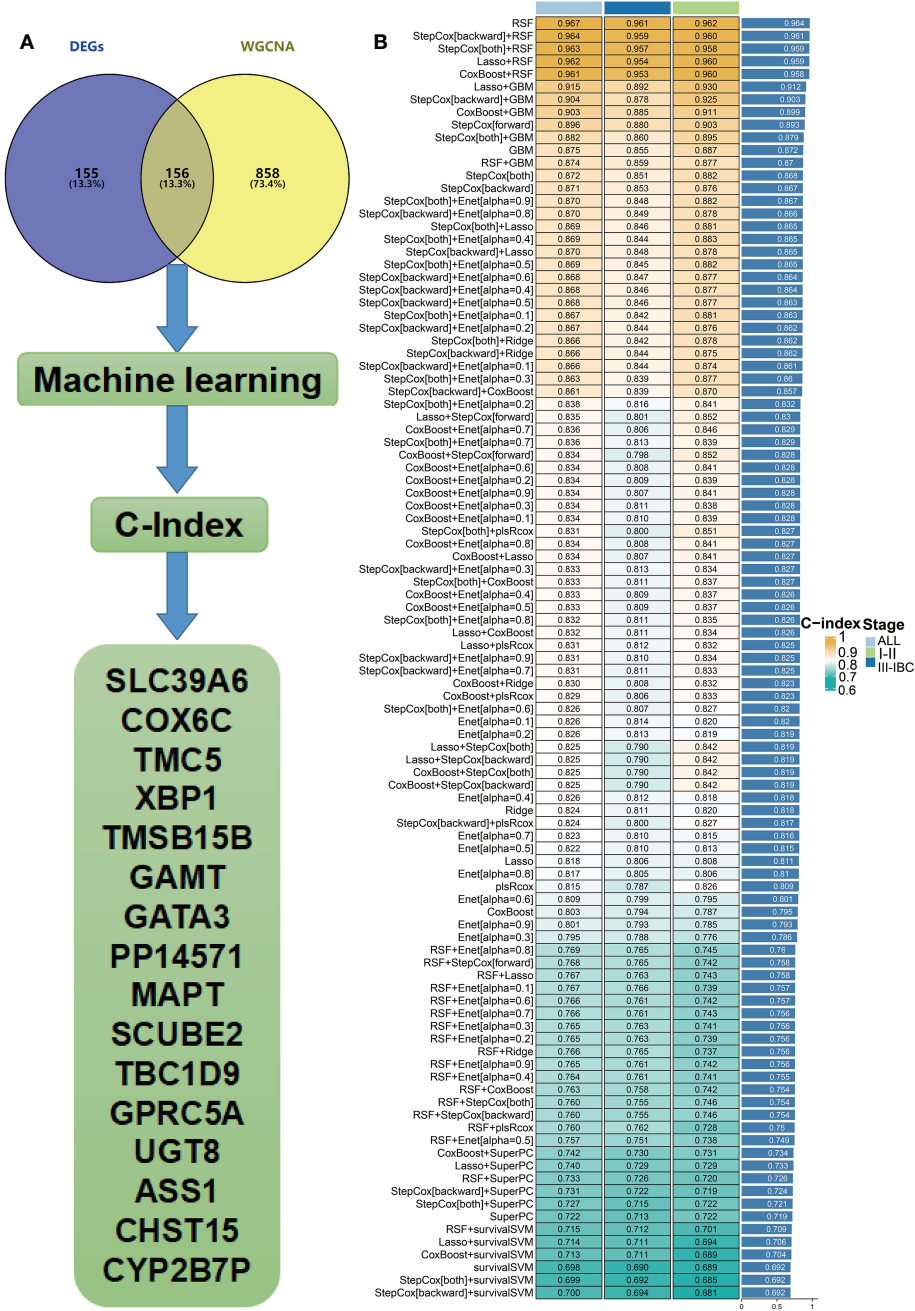
Hub genes were screened within machine learning. **(A)** Flow chart describing the screen for RCB-related genes impacting the DRFS of BC patients after NAC. **(B)** Performance comparison of prediction models based on different machine learning methods (IBC : Inflammatory breast cancer).

We intersected the module genes obtained from WGCNA with the DEGs acquired through limma analysis, resulting in 156 RCB-related genes (**Figure 4A**). These 156 RCB-related genes underwent a machine learning-based integrative procedure to further refine the core gene signatures that significantly influence the DRFS of BC. Within the GSE25066 cohort, we employed the leave-one-out cross-validation (LOOCV) framework to build 101 prediction models and calculated the C-index for each model across various American Joint Committee on Cancer (AJCC) stages. Consequently, the optimal model was the random survival forest (RSF), exhibiting the highest average C-index (0.964), which outperformed all other models across different AJCC stages (**Figure 4B**). Employing the RSF algorithm, we pinpointed a total of 16 RCB-related gene signatures that impact DRFS (**Supplementary Table 5**). Among these signatures, TMSB15B, UGT8, and ASS1 displayed elevated expression in the RCB0/I group, while the other genes exhibited higher expression in the RCBII/III group (**Supplementary Figure 1A**).

### A newly developed molecular subtyping to predict the DRFS of BC

**Figure 5:**
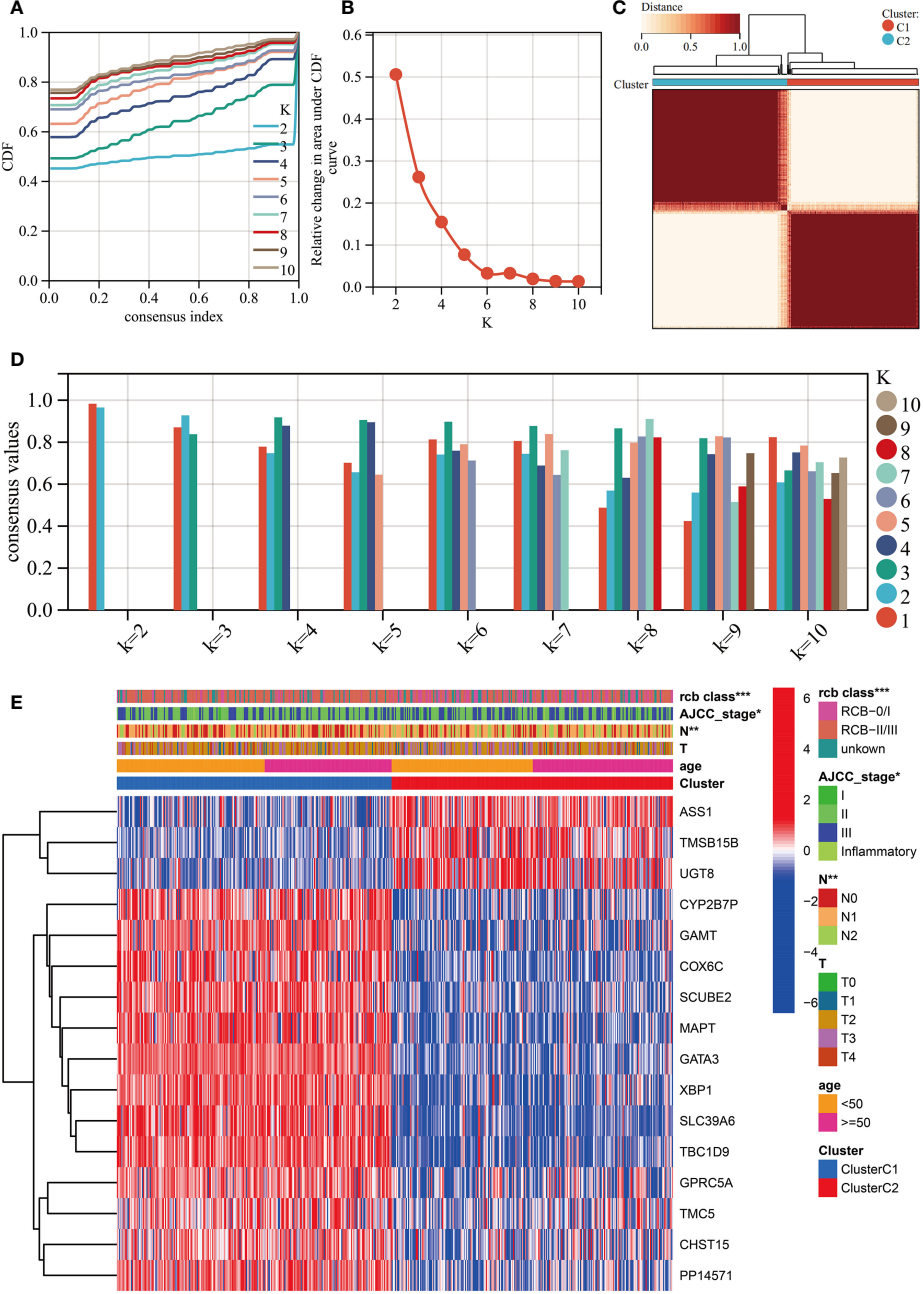
Unsupervised clustering performed in training dataset (GSE25066). **(A)** Consensus clustering cumulative distribution function (CDF) for k = 2-10. **(B)** Relative change in the area under the CDF curve (k = 2-10). **(C)** Consensus clustering matrix for k=2. **(D)** Cluster consensus values for k = 2-10. **(E)** Heatmap for the normalized expression of the 16 RCB-related gene signatures. (*: *p*<0.05, **: *p*<0.01, ***: *p*<0.001).

**Figure 6:**
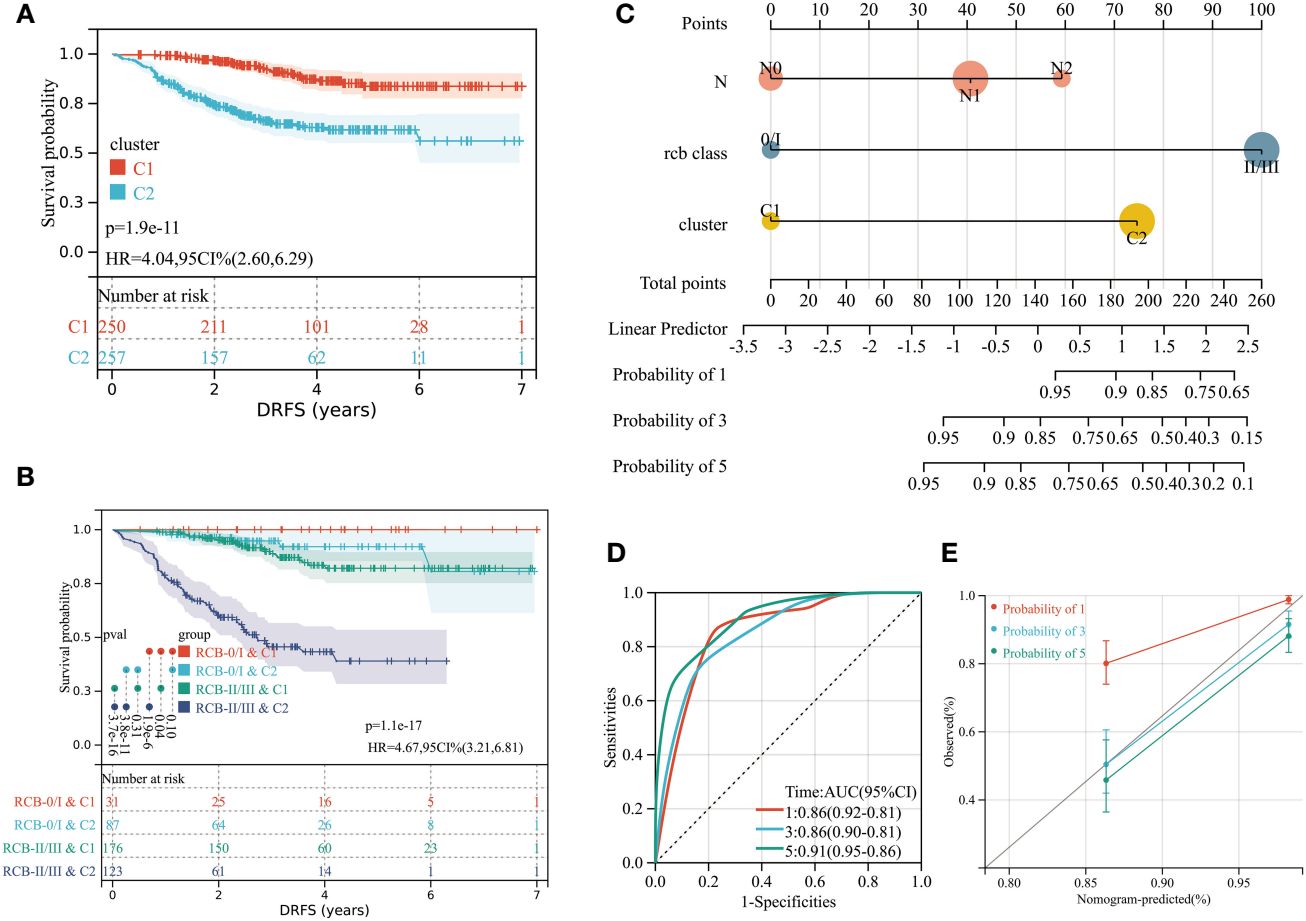
**(A)** Differences in DRFS between different molecular subtypes in training dataset (GSE25066). **(B)** KM curve analysis of DRFS is shown for patients classified according to molecular subtype and RCB class. **(C)** Developed nomogram. The nomogram was developed with the N stage, RCB class and molecular subtype. **(D)** ROC curve demonstrating diagnostic performance of nomograms for DRFS. **(E)** The calibration curve to evaluate the accuracy of the nomogram at 1, 3 and 5 years, respectively.

We performed unsupervised clustering using the R package “ConsensusCluster Plus” on the 16 RCB-related gene signatures in GSE25066. The ideal cluster number was identified based on the empirical cumulative distribution function (CDF) plot (**Figures 5A, B**), with the best partition efficiency achieved at k = 2 based on consensus scores (**Figures 5C, D**). As a result, we divided BC patients into different molecular subtypes (cluster C1 & cluster C2), and the heat-map indicated distinct gene expression patterns of the 16 RCB-related gene signatures between the clusters (**Figure 5E**). Notably, patients in cluster C2 had a poorer DRFS than those in cluster C1 (HR: 4.04; 95% CI: 2.60–6.29; log-rank test p<0.0001; **Figure 6A**). We investigated whether the DRFS differences were attributed to RDB and found no significant difference in the proportion of RDB III patients between the clusters (27% vs. 26%), but a greater proportion of patients who achieved a pathological complete response (pCR) after NAC were observed in cluster C1 (**Supplementary Figure 1C**). To further validate the predictive capability of the novel molecular subtyping scheme for DRFS, multivariate analysis was performed and showed that our molecular subtyping scheme was an independent strong predictor for DFRS in BC (HR: 5.20; 95% CI: 3.13–8.60; p<0.0001) (**Supplementary Figure 1D**).

A stratified analysis of the prognosis for DRFS based on the RCB index and pathologic response was performed (**Supplementary Figure 2**), which isolated patients with greater risk of distant recurrence from the RCB II, RCB III, and residual disease (RD) subcategories based on the molecular subtyping scheme. We investigated the possible reasons for the better DRFS of cluster C1, which had fewer pCR patients. CIBERSORT algorithm analysis indicated that 16 types of immune cells were significantly differently infiltrated in different clusters (**Supplementary Figure 3A**), with the most significant difference being the higher number of resting mast cells in cluster C1 (p<0.0001). Furthermore, KM analysis revealed that higher resting mast cell infiltration is a significant protective factor of distant recurrence for BC patients with NAC (HR: 0.42; 95% CI: 0.25–0.71; log-rank test p=0.00087; **Supplementary Figure 3C**). The vast majority (96%) of cluster C1 patients had estrogen receptor (ER)-positive tumors, which were observed rarer (19%) in pCR patients. In summary, resting mast cell infiltration and ER status may contribute to the different risks for distant recurrence between cluster C1 and C2.

Incorporating the RCB index with the molecular subtyping scheme could improve the predictive accuracy of DRFS before the start of NAC (**Figure 6B**). Within the overall follow-up time (mean: 3.76 years), no patients experienced a distant recurrence in the “RCB 0/I & C1” group. The “RCB I/II & C2” subcategory had the poorest prognosis in terms of DRFS, with the median time to distant recurrence being 2.52 years. To aid in clinical use, a nomogram was established based on the independent prognostic factors (N stage, RCB class, molecular subtyping) identified by multivariate analyses (**Figure 6C**).To evaluate the accuracy of the nomogram, we drew a ROC curve and calibration plot (**Figures 6D, E**). The ROC curve analysis for the 5-year DRFS demonstrated an outstanding predictive performance (AUC = 0.91, 95% CI: 0.95–0.86; C-index = 0.85, 95% CI: 0.81–0.90).

### Independent validation of molecular subtyping scheme

We conducted unsupervised clustering using the independent validation set (GSE32603) and achieved optimal partition efficiency with k=2, based on consensus scores and CDF curves (**Supplementary Figures 3B, 4A, C, D**). The heatmap depicted a highly similar gene expression pattern of the 16 RCB-related gene signatures between GSE25066 and GSE32603 (**Supplementary Figure 4E**). Clinical characteristics were comparable between different clusters in the validation set and the training dataset (**Supplementary Figures 5A, B**). Patients within cluster C2 exhibited inferior RFS compared to those in cluster C1 (HR: 2.11; 95% CI: 1.10–4.02; log-rank test p=0.02; **Supplementary Figure 5C**). Integration of the RCB index with the molecular subtyping scheme in the validation set enhanced the predictive accuracy of RFS estimation before the commencement of NAC (**Supplementary Figure 5D**). Therefore, our molecular subtyping scheme was regarded as appropriate and generalizable.

### GPSA analysis reveals key gene associated with RCB

**Figure 7:**
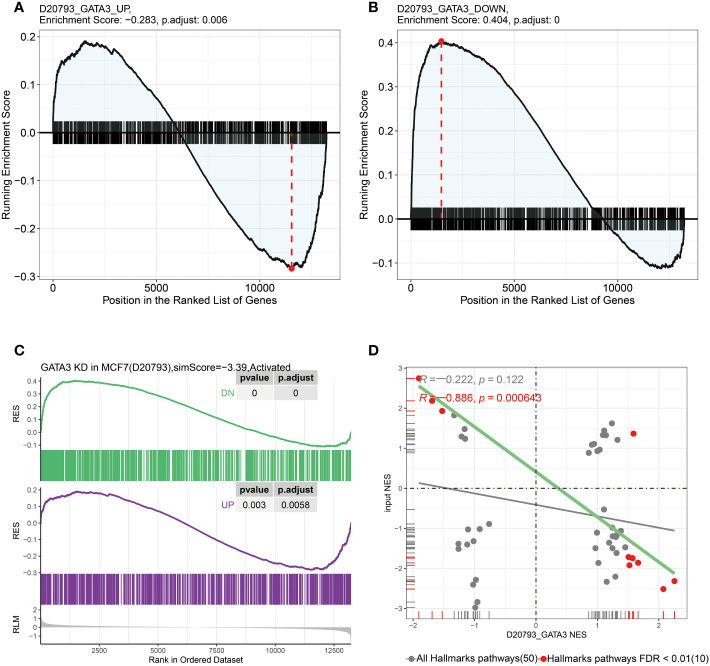
In GPSA database, the up-regulated and down-regulated gene sets were obtained by differentially expressed gene analysis after GATA3 knocked down using shRNA. **(A)** The up-regulated gene set were enriched in RCB 0/I group and **(B)** the down-regulated gene set were enriched in RCB II/III group. **(C)** The pattern of gene expression in RCB II/III group was opposite to that observed in the MCF7 cell lines with knockdown of GATA3. **(D)** The results of pearson correlation analysis between the NES of hallmark gene sets enriched in both RCB II/III group and GATA3 shRNA knocked down cell line (R=-0.886, *p* < 0.001).

To identify the key genes associated with RCB, we performed GPSA analysis on differentially expressed genes between the RCB 0/I and RCB II/III groups. We identified 327 genes that shared similar downstream effects with the input data when knocked down/out (**Supplementary Table 6**). The intersection of these 327 genes with the 16 RCB-related gene signatures affecting DRFS identified by the RSF algorithm resulted in the identification of GATA3 as the key gene associated with RCB. The pattern of gene expression in the RCB II/III group was opposite to that observed in MCF7 cell lines with knockdown of GATA3 (**Figures 7A–C**). There was a strong negative correlation between the enrichments in the Hallmarks pathways of the RCB II/III group and the MCF7 cell lines with knockdown of GATA3 (R= -0.886, p<0.0001; **Figure 7D**). These results provide strong evidence supporting the rationale of GATA3 as the key gene associated with RCB.

### Relationship between GATA3 and mast cells revealed by single-cell analyses

**Figure 8:**
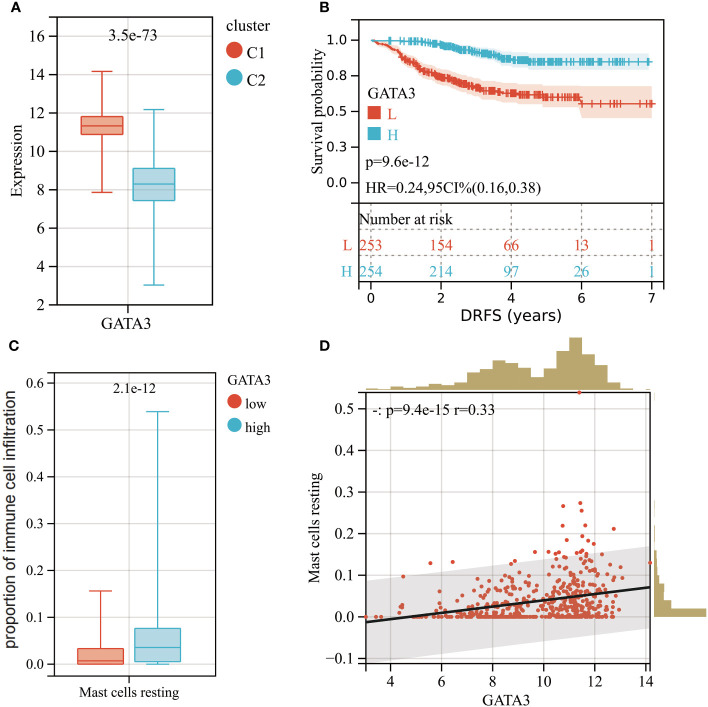
**(A)** Boxplot of GATA3 expression levels between different molecular subtypes (Red: Cluster C1; Blue: Cluster C2). **(B)** KM analysis demonstrating the difference of DRFS between low and high levels of GATA3. **(C)** Box plot showed the differences in infiltration levels of resting mast cells between low and high levels of GATA3 group. **(D)** Spearman correlation analysis between the expression level of GATA3 and resting mast cells infiltration.

GATA3 expression was found to be higher in cluster C1, which had a better DRFS (**Figure 8A**). High GATA3 expression was positively correlated with better DRFS in BC patients after NAC (HR: 0.24; 95% CI: 0.16–0.39; p<0.0001; **Figure 8B**). Furthermore, BC tumors with high GATA3 expression showed greater infiltration of resting mast cells (**Figure 8C**). Spearman analysis demonstrated a significant positive correlation between GATA3 expression and the extent of tumor-infiltrating resting mast cells in the tumors (R= 0.33, p<0.0001; **Figure 8D**).

**Figure 9:**
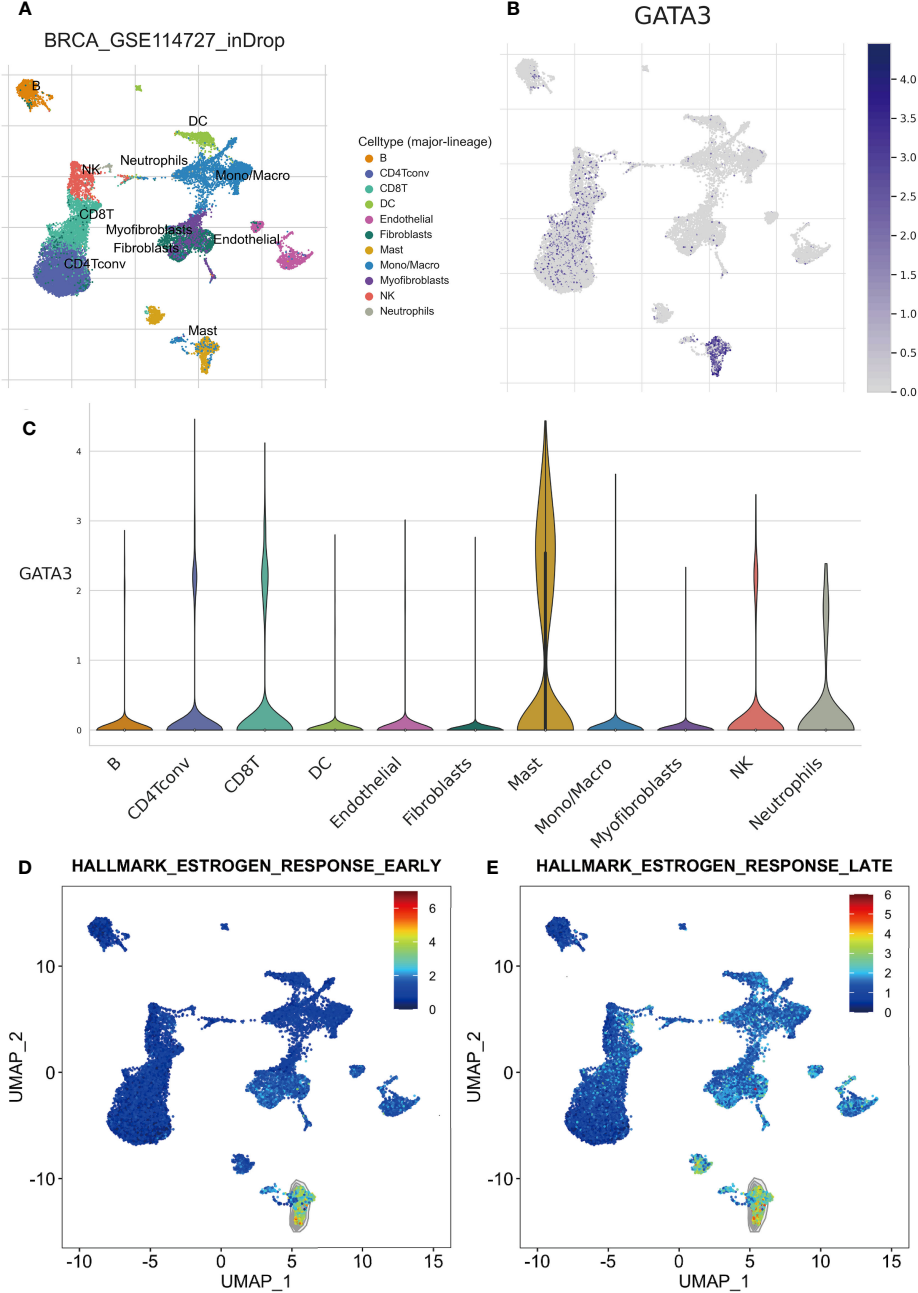
Results of scRNA-seq analysis basedon TISCH database. **(A)** UMAP plot of all the single cells in GSE114727-inDrop cohort, with each color coded for 11 major cell types. **(B)** Relative expression of GATA3 genes in distinct cell types (the bluer the color, the higher the expression). **(C)** Violin plots showing the expression of GATA3 genes in distinct cell types. **(D)** The degree of enrichment of “Hallmark-Estrogen Response Early” and “Hallmark-Estrogen Response Late”pathways in different cell types (the redder the color, the higher the degree).

To explore the connection between mast cell infiltration and GATA3 within tumor tissue, we conducted single-cell sequencing analysis and employed the TISCH database. A total of 11 cell types were identified in the GSE114727 (in-Drop) dataset, including B cells, CD8 T cells, CD4 Tconv cells, endothelial cells, fibroblasts, mast cells, dendritic cells, macrophages, myofibroblasts, natural killer (NK) cells, and neutrophils (**Figure 9A**). Intriguingly, GATA3 exhibited the highest expression level in mast cells infiltrating BC tumors, consistent with our prior findings (**Figures 9B, C**). Additionally, the “Hallmark-Estrogen Response Early” and “Hallmark-Estrogen Response Late” pathways were particularly enriched in mast cells (**Figures 9D, E**), indicating that mast cells possess a heightened capacity to respond to estrogen compared to other immune cells infiltrating BC tumors.

### Discovery of potential drugs by computational methods

**Figure 10:**
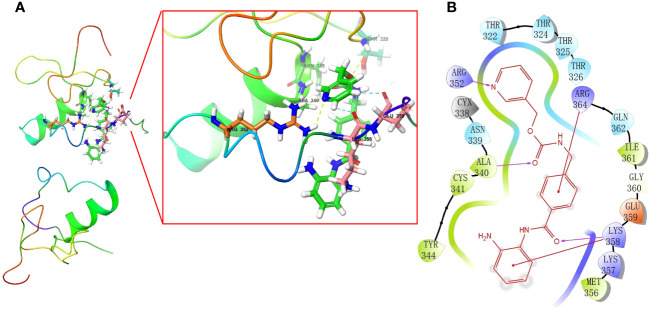
In silico molecular docking of GATA3 protein with Entinostat using Schrodinger software. Schematic 3D **(A)** and 2D **(B)** representation of molecular docking models, active sites, and binding distances.

In our study, we used the “XSum” algorithm to perform CMap analysis with the top 1000 DEGs (500 up-regulated and 500 down-regulated genes) between RCB 0/I and RCB II/III groups as input. Our analysis revealed that Entinostat (MS-275) had the minimum XSum score (**Supplementary Table 7**), indicating that it is a potential small molecular compound to reverse high RCB after NAC. In other words, Entinostat has the potential to reduce tumor burden and control residual tumors after NAC. To explore the possibility of Entinostat acting as a direct GATA3 inhibitor, we conducted molecular docking analyses using Schrodinger software. Three-dimensional and two-dimensional docking poses of Entinostat and GATA3 protein were shown in **Figures 10A**, **B**, respectively. Our findings suggest that Entinostat has a favorable binding affinity for the GATA3 protein, as evidenced by a docking glide score of -7.573 kcal/mol. Therefore, Entinostat may represent a novel and promising strategy for increasing the efficacy of NAC.

### Exploration of EDC exposures with potential to impact the DRFS of BC

We explored all potential EDC Exposures that may impact the expression levels of the 16 Key Genes Associated with RCB and DRFS by leveraging the CTD database. Subsequently, we have acquired a total of 19 different types of EDC Exposures that could affect the expression level or methylation state of the 16 Key Genes, showing in **Supplementary Table 9**. Thus, these EDC Exposures have the potential to modulate the RCB and DRFS of BC, an effect that is mediated by the intermediary factors of these 16 Key Genes. Hence, avoiding exposure to these EDCs may facilitate an improvement in the effectiveness of NAC among BC patients. Further studies may be necessary to elucidate the underlying mechanisms and ultimately improve outcomes in the management of BC.

### Relationship between molecular subtyping strategies and tumor metabolism and cellular senescence

The enrichment levels of pathways related to tumor metabolism and cellular senescence were evaluated across distinct clusters of patients using the ssGSEA method. The Wilcoxon rank sum test revealed a significant difference in the enrichment scores of pathways related to metabolism and cellular senescence between Cluster 1 and Cluster 2 (**Supplementary Table 10**). Out of the 15 pathways associated with cellular senescence, 11 displayed significantly higher enrichment scores in Cluster 2 compared to Cluster 1. The “reactome oncogene induced senescence” pathway exhibited the most significant difference in enrichment scores between clusters. Significant differences were observed between the metabolic profiles of Cluster 1 and Cluster 2 (**Supplementary Figure 6A**). Cluster 1 exhibited elevated levels of fatty acid metabolism, propanoate metabolism, ascorbate and aldarate metabolism, and butanoate metabolism, while Cluster 2 demonstrated increased levels of galactose metabolism. We also evaluated the association between the key gene, GATA3, and both metabolism and cellular senescence (**Supplementary Figure 6B**). Metabolic pathways, including alanine aspartate and glutamate metabolism, ascorbate and aldarate metabolism, beta alanine metabolism, butanoate metabolism, and fatty acid metabolism, were found to be significantly positively associated with GATA3 gene expression levels (**Supplementary Figure 6B**). Most cellular senescence pathways were found to be enriched with a negative correlation with GATA3 gene expression levels. However, the sphingolipid metabolism in senescence pathway showed a significant positive correlation with GATA3 gene expression levels (**Supplementary Figure 6C**).

### Experimental validation of GATA3 as a biomarker for BC and RCB after NAC

**Figure 11:**
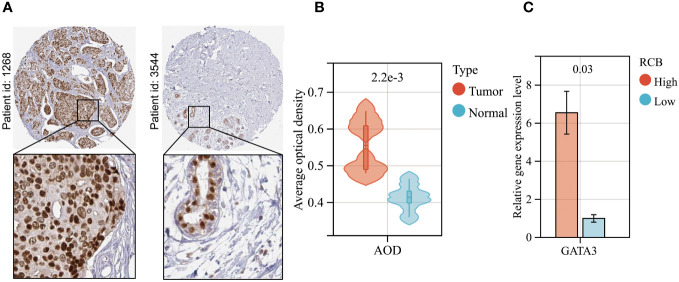
Validation of GATA3 expression levels. **(A)** GATA3 protein expression in immunohistochemical images of BC (left) and normal (right) samples. **(B)** The AOD of GATA3 protein in BC (Red) and normal (Green) samples. **(C)** Results of PCR analysis.

To begin with, high-definition immunohistochemical images from the HPA database were retrieved for breast cancer and normal breast tissues. These images were utilized to assess the differential GATA3 protein expression levels between the two tissue types, using an AOD evaluation method (**Figure 11A**). There is a significant upregulation of GATA3 protein expression levels in breast cancer tissues (**Figure 11B**). We collected post-operative specimens from eight breast cancer patients subjected to neoadjuvant taxane-anthracycline chemotherapy. Tumor diameter measurements were taken before chemotherapy, and the remaining tumor load was determined from post-operative pathology reports. Of the eight patients, four had tumors that shrunk more than 80% after chemotherapy and were classified into the RCB-Low group. On the other hand, the remaining four patients with tumors that shrank less than 50% after chemotherapy were classified into the RCB-High group. Our findings from PCR assays showed that GATA3 gene expression levels were significantly upregulated in the RCB-High group, in agreement with the previous bioinformatics analysis results (**Figure 11C**).

### Meta-analysis validation of GATA3 as a predictive biomarker for BC tumor recurrence

**Figure 12:**
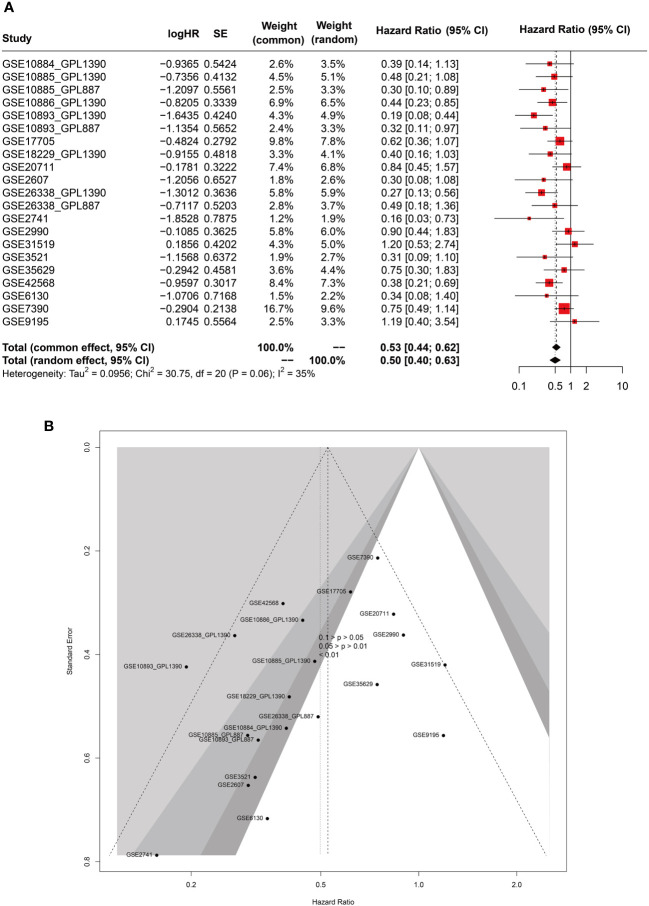
Forest **(A)** and funnel **(B)** plots of meta-analysis on GATA3 and RFS of BC.

In this Meta-analysis, a total of 21 cohorts were included. It merits mentioning that even microarray datasets derived from the same study but measured on distinct platforms (GPL) were regarded as separate cohorts, given the batch effect of the gene sequencing. Both the common effect model (HR=0.53, 95%CI [0.44-0.62], Z=-7.35, *p*<0.0001) and random effects model (HR=0.50, 95%CI [0.40-0.63], Z=-5.99, *p*<0.0001) demonstrated GATA3 as a favorable protective factor against BC recurrence. The heterogeneity among the included studies was deemed acceptable (P=0.06, I2 = 35%). Accordingly, we opted for the results derived from the random effects model (**Figure 12A**). Our findings from the Eggers and Beggs tests indicated that there was no significant publication bias (Eggers test, *p*= 0.0534; Beggs test, *p*=0.07) (**Figure 12B**). In conclusion, our meta-analysis results corroborated the findings from our bioinformatics analysis, indicating GATA3 as a protective factor against breast cancer recurrence.

### Results of MR analysis

**Figure 13:**
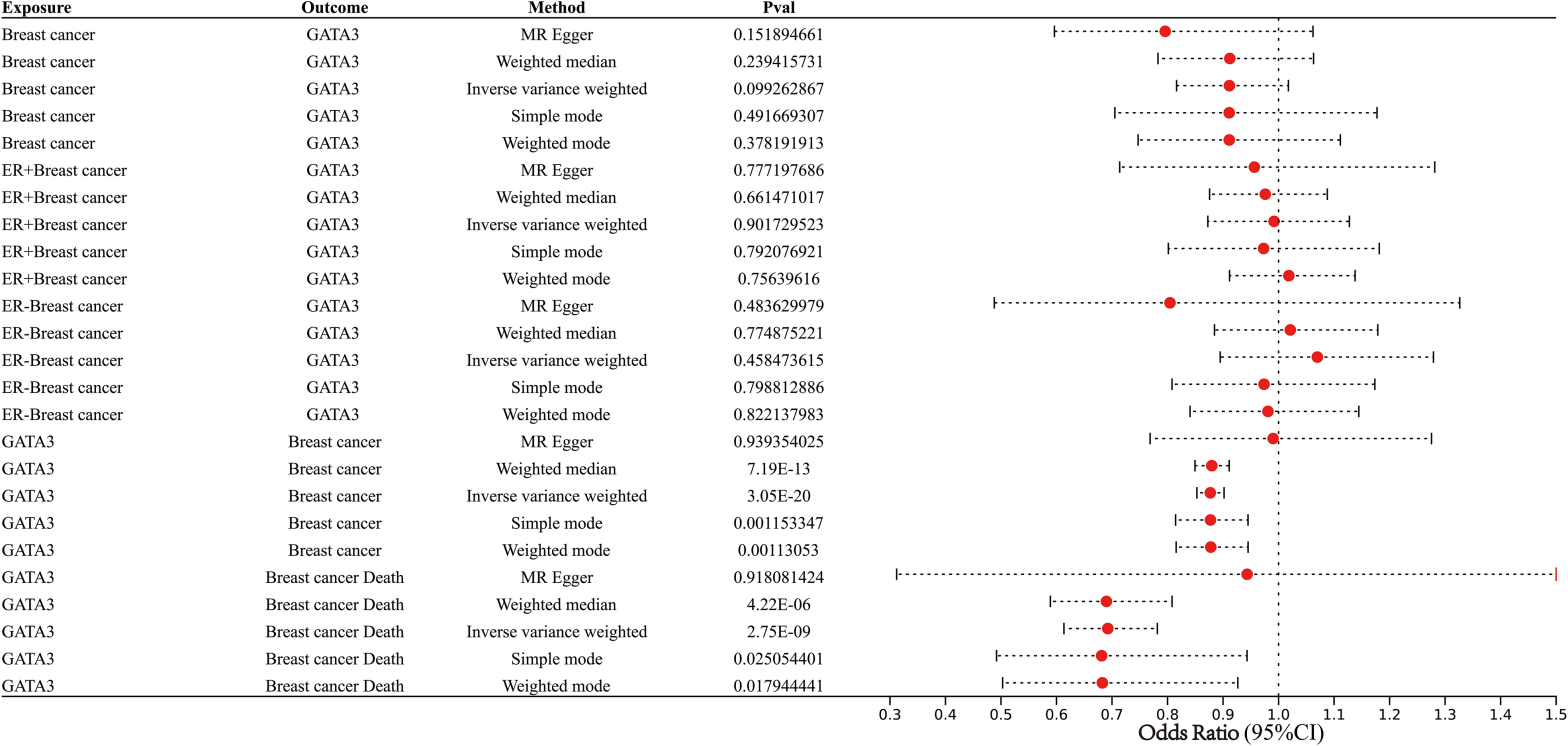
Forest plot showing results from the Mendelian randomization analysis.

MR integrated GWAS with eQTL data to test for the association BC and eQTL of GATA3 (**Figure 13**; **Supplementary Figure 7**). The results from the IVW, WM, MR-Egger methods, simple mode, and weighted model analyses collectively suggest that changes in GATA3 gene expression are not a causative factor for BC occurrence (including ER+ and ER- subtypes). Instead, mutations in GATA3 eQTL (leading to downregulation of the GATA3 gene) could serve as protective factors against the occurrence of BC (IVW: *p*<0.001, WM: *p*<0.001, simple mode: *p*=0.001, and weighted model: *p*=0.001). There is a causal relationship between downregulation of the GATA3 gene and breast cancer mortality (IVW: *p*<0.001, WM: *p*<0.001, simple mode: *p*=0.03, and weighted model: *p*=0.02).

## Discuss

RCB is a highly intuitive indicator for assessing the effectiveness of NAC regimens and has also found utility in predicting clinical outcomes in BC patients (22). Nevertheless, several studies have indicated that even patients achieving pCR after preoperative or postoperative chemotherapy might not experience complete recovery or local control, with some of these tumors still at risk of recurrence (23–25). As a result, relying solely on RCB to predict DRFS following NAC is inadequate. There is an urgent demand for a novel and robust classifier to accurately predict individual risks of distant recurrence in clinical settings.

In this study, we investigated disparities in gene expression patterns and the activation of signaling pathways between high and low levels of RCB after NAC treatment. Utilizing a combination of machine learning models and WGCNA, we identified significant gene signatures closely linked to RCB. Additionally, the GPSA database facilitated the analysis of multiple knockdown studies, ultimately pinpointing GATA3 as a pivotal gene signature associated with RCB after NAC treatment. Among the most frequently mutated genes in BC, GATA3 (11%) took precedence, followed by TP53 and PIK3CA (26). GATA3, along with its downstream target FOXA1, assumes a critical role in upholding the luminal differentiation status of human mammary epithelial cells (27, 28). Furthermore, GATA3 acts as a constraint on the metastatic dispersion of tumor cells by impeding the epithelial-to-mesenchymal transition (EMT) process (29). This, in part, elucidates the connection between GATA3 and improved DRFS observed in our investigation (**Figure 8B**). Moreover, our research employed an array of validation techniques, including IHC, Rt-PCR, meta-analysis, and MR, to corroborate our bioinformatic analysis findings. Initial validation through IHC and RT-PCR established the association of GATA3 expression levels with both BC development and RCB progression subsequent to neoadjuvant therapy. Although our preliminary results suggest that GATA3 is a biomarker for DRFS in breast cancer patients following NAC treatment, there are limited applicable datasets with DRFS information of BC patients in the GEO database. Therefore, we expanded our search and conducted a meta-analysis to investigate the potential of GATA3 as a biomarker for RFS (including DRFS) in BC patients. Then, meta-analysis of 21 independent cohorts confirmed the close relationship between GATA3 and RFS of BC. The use of MR enabled us to explore the causality between GATA3 and breast cancer incidence and prognosis. Our comprehensive validation, involving large-scale cohorts, was consistent with the results of our bioinformatic analysis.

Furthermore, prior studies have demonstrated that concurrent expression of GATA3 and Hes-1 skews the cell fate of myeloid progenitors toward downstream progenitors capable of generating mast cells at the single-cell level (30). In our investigation, there was a significant positive correlation between resting mast cells and GATA3 expression levels in BC. According to Xie et al., tumor-infiltrating mast cells can mitigate the efficacy of chemotherapy and radiotherapy by influencing the p38/p53/p21 signaling pathway and ATM phosphorylation (31). This could partly elucidate why tumors with higher levels of resting mast cell infiltration, such as those in cluster C1 and the RCBII/III group, exhibit more residual tumors post NAC. Furthermore, Majorini et al. explored whether co-culturing with mast cells impacts the expression of ER in various panels of human and mouse BC cell lines (32). In all tested cell lines, the presence of mast cells led to a significant increase in ER transcription and protein levels. Our single-cell sequencing analysis findings also supported this observation. ER-positive BC, generally, carries a more favorable prognosis compared to ER-negative tumors, exhibiting reduced aggressiveness, with its development and progression regulated by ER (33). A study involving a sizable sample revealed that ER-positive BC carries an extended risk of recurrence, with around 50% of recurrences transpiring after 5 years (late distant recurrence, LDR), in contrast to ER-negative BC, which primarily recurs within the initial 5 years (34). Additionally, patients with ER-positive BC can gain benefits from prolonged endocrine treatment, a strategy proven to further curtail the risk of both local and distant recurrence (35). In essence, manipulation of the GATA3-mast cell-ER axis may hold promise as a prospective therapeutic target to mitigate the risk of distant recurrence and enhance outcomes for BC patients. Moreover, the GATA3-mast cell-ER axis forms the underlying biological foundation for our devised molecular subtyping scheme, aiming to predict DRFS after NAC.

In our study, we developed an mRNA expression-based molecular subtyping scheme and a nomogram to predict distant DRFS in BC patients following NAC. Our nomogram demonstrated excellent prediction ability, with an AUC of 0.91 for 5-year DRFS, outperforming many previous prediction tools (**Supplementary Table 8**) (36–45). Importantly, our study used microarray data collected prior to NAC treatment, suggesting that our results have the potential to guide clinical decision-making, particularly before NAC treatment initiation. If a BC patient is identified as high risk for distant tumor recurrence by our nomogram, further adjuvant therapies and close monitoring are required to prevent and detect relapse.

Through bioinformatic technologies, we identified Entinostat as a potential therapeutic drug to further reduce RCB when combined with NAC. Entinostat is an oral synthetic benzamide-derivative that inhibits HDAC1 and HDAC3 enzymes, and has shown promising antitumor activity *in vitro* and *in vivo* (46–58). Combination therapy with chemotherapeutic agents and Entinostat has been shown to enhance anti-proliferative activity and overcome treatment resistance in preclinical researches. Safety evaluations of Entinostat for BC patients in a Phase III Clinical Trial indicated relatively low levels of adverse events, similar to previous research (57, 58) However, the effects of Entinostat on the efficacy of NAC in BC patients have not been reported, warranting further investigation.

We present an extensive review with the aim of investigating the impact of exposure to endocrine-disrupting chemicals (EDCs) on the expression levels of RCB-related genes - a phenomenon that may potentially influence the disease-free survival (DRFS) of breast cancer (BC) following neoadjuvant chemotherapy (NAC). Our objective is to illuminate the pivotal interplay between external factors and NAC, along with its clinical implications within the context of BC pathogenesis. Through our research, we offer fresh insights and resources that can facilitate a more comprehensive exploration of the intricate relationship between BC progression and exposure to EDCs. Consequently, these findings hold the potential to offer new perspectives for guiding clinical treatment strategies for BC patients, ultimately enhancing the standard of care for this condition.

Although our study provides novel insights into optimizing therapeutic and surveillance regimens for distant recurrence after NAC, there are still some limitations that need to be acknowledged, such as the reliance on association studies and bioinformatics analysis. Further experimental studies based on the observations of the current study are required. Our findings may improve targeted prevention and personalized treatment strategies in BC, leading to a paradigm shift from reactive medical services to predictive, preventive, and personalized medicine. Overall, the current study aims to identify a potential biomarker to predict DRFS after NAC, which could increase the efficiency of NAC and reduce treatment costs.

## Conclusions

Based on combination of bioinformatics and machine learning analysis, we fully explore the difference of gene expression pattern and activation of signaling pathways between high and low level of RCB after NAC treatment. Furthermore, multiple knockdown studies were analyzed by GPSA database and then GATA3 was further screened out as a key gene signature of RCB following NAC. Subsequently, we constructed and verified a mRNA expression-based molecular subtyping scheme and a nomogram, which were able to accurately predict DRFS in BC patient following NAC. This molecular subtyping scheme was found to be closely associated with tumor metabolism and cellular senescence. The GATA3-mast cell-ER axis is also the potential biological basis for the our molecular subtyping scheme established to predict DRFS after NAC. We also provided a comprehensive review of the EDCs exposures that potentially impact the effectiveness of NAC among BC patients. Our study contributes to the optimization of personalised clinical management and treatment regimens of BC.

## Data availability statement

The original contributions presented in the study are included in the article/**Supplementary Material**, Further inquiries can be directed to the corresponding authors.

## Ethics statement

The studies involving humans were approved by The Second Affiliated Hospital of Anhui Medical University. The studies were conducted in accordance with the local legislation and institutional requirements. The participants provided their written informed consent to participate in this study.

## Author contributions

JH: Formal Analysis, Investigation, Methodology, Writing – original draft, Writing – review & editing. JZ: Data curation, Formal Analysis, Resources, Conceptualization, Writing – review & editing. LA: Methodology, Project administration, Software, Investigation, Writing – review & editing. ML: Data curation, Supervision, Validation, Investigation, Writing – review & editing. MZ: Validation, Visualization, Writing – review & editing, Writing – original draft. YW: Formal Analysis, Methodology, Visualization, Writing – review & editing, Investigation, Writing – original draft. QW: Funding acquisition, Supervision, Writing – review & editing, Writing – original draft.

## Supplementary material

Supplementary Figure 1
**(A)** Boxplot of the expression levels of the 16 RCB-related gene signatures between different RCB classes (Red: RCB II/III; Blue: RCB 0/I). **(B)** The proportion of BC patients with different RCB classes in Cluster C1 and Cluster C1 (Blue: RCB III; Red: RCB II; Green: RCB 0/I). **(B)** The proportion of BC patients with different pathologic response in Cluster C1 and Cluster C1 (Blue: RD; Red: pCR). **(C)** Multivariate analysis for risk factor for distant recurrence of BC patients after NAC.

Supplementary Figure 2 KM curves showing the difference of DRFS in Cluster C1 and Cluster C2 stratified by RCB class and pathologic response.

Supplementary Figure 3
**(A)** Boxplot showing the infiltration levels of 22 immune cell types in different molecular subtypes (Red: Cluster C1; Blue: Cluster C2). **(B)** Box plot showing the differences in infiltration levels of resting mast cells between different RCB class (Red: RCB 0/I; Green: RCB II/III). **(C)** KM analysis demonstrating the difference of DRFS between low and high infiltration levels of resting mast cells. **(D)** The proportion of BC patients with different ER status in Cluster C1 and Cluster C1 (Blue: positive; Red: negative). **(E)** The proportion of BC patients with different ER status in RD group and pCR group (Blue: positive; Red: negative).

Supplementary Figure 4 Unsupervised clustering performed in verification dataset (GSE32603). **(A)** Consensus clustering cumulative distribution function (CDF) for k = 2-10. **(B)** Relative change in the area under the CDF curve (k = 2-10). **(C)** Consensus clustering matrix for k=2. **(F)** Cluster consensus values for k = 2-10. **(D)** Heatmap for the normalized expression of the 16 RCB-related gene signatures.

Supplementary Figure 5 Verification of the predictive ability of our molecular subtyping scheme in verification dataset (GSE32603). **(A)** The proportion of BC patients with different RCB class in Cluster C1 and Cluster C1 (Blue: RCB 0/I; Red: RCB II/III). **(E)** The proportion of BC patients achieved pCR after NAC in Cluster C1 and Cluster C1 (Blue: pCR-No; Red: pCR-Yes). **(A)** Differences in RFS between different molecular subtypes in GSE32603. **(B)** KM curve analysis of RFS is shown for patients classified according to molecular subtype and RCB class in GSE32603.

Supplementary Figure 6
**(A)** The heatmap displays the enrichment scores of the metabolism and senescence-related pathways between Cluster 1 and Cluster 2. The results of the Spearman correlation analysis between the expression level of GATA3 gene and the enrichment scores of the metabolism **(B)** and senescence **(C)** -related pathways.

Supplementary Figure 7 The scatter plot of five MR methods.
