# Supplementary figures and images for: Proposing a novel molecular subtyping scheme for predicting distant recurrence-free survival in breast cancer post-neoadjuvant chemotherapy with close correlation to metabolism and senescence
Source: Front Endocrinol (Lausanne). 2023 Oct 12;14:1265520. doi: 10.3389/fendo.2023.1265520 (PMC10602753; doi:10.3389/fendo.2023.1265520)

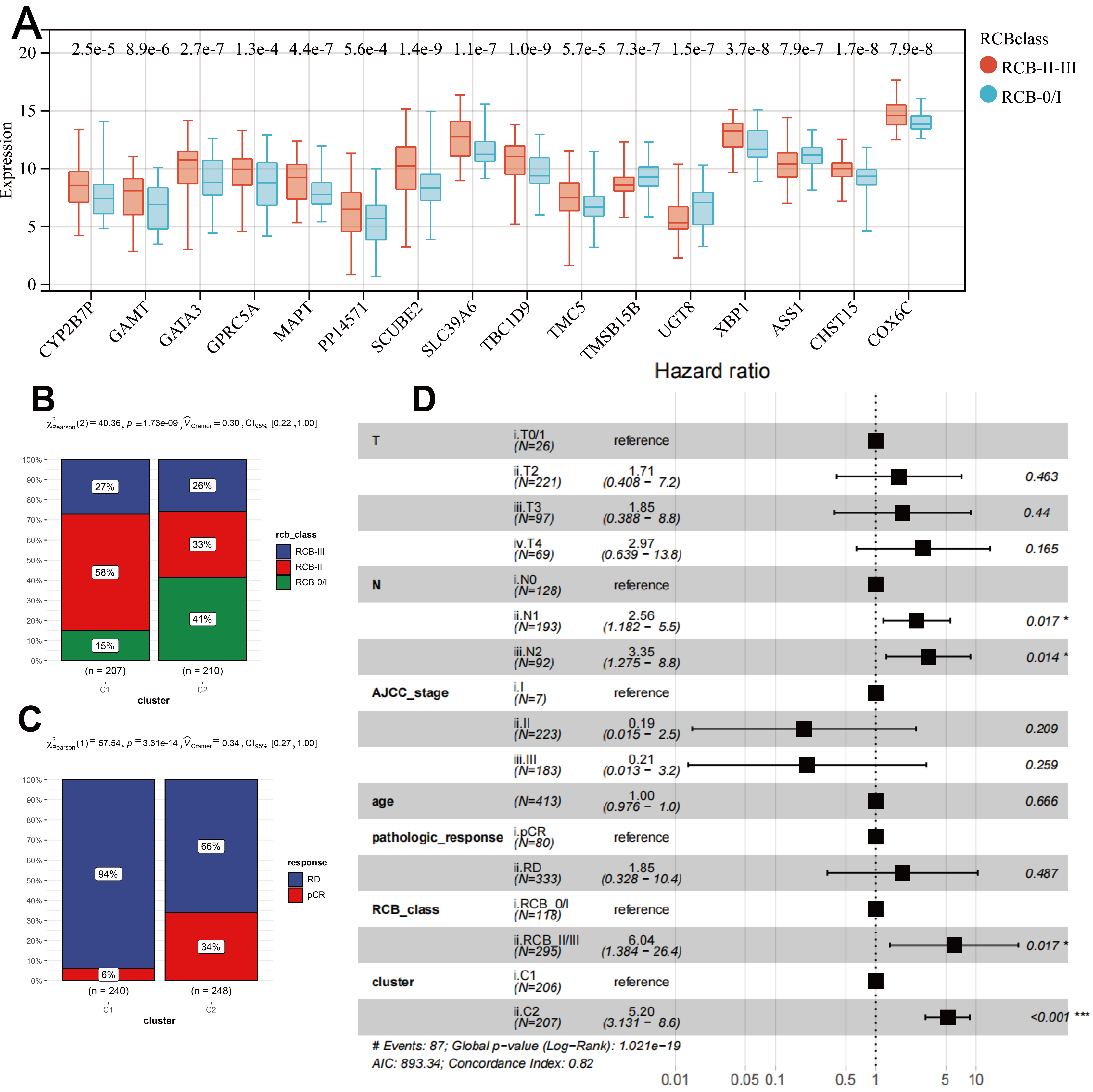

Supplement: Supplementary Figure 1 — (A) Boxplot of the expression levels of the 16 RCB-related gene signatures between different RCB classes (Red: RCB II/III; Blue: RCB 0/I). (B) The proportion of BC patients with different RCB classes in Cluster C1 and Cluster C1 (Blue: RCB III; Red: RCB II; Green: RCB 0/I). (B) The proportion of BC patients with different pathologic response in Cluster C1 and Cluster C1 (Blue: RD; Red: pCR). (C) Multivariate analysis for risk factor for distant recurrence of BC patients after NAC. [file Image_1.jpeg]

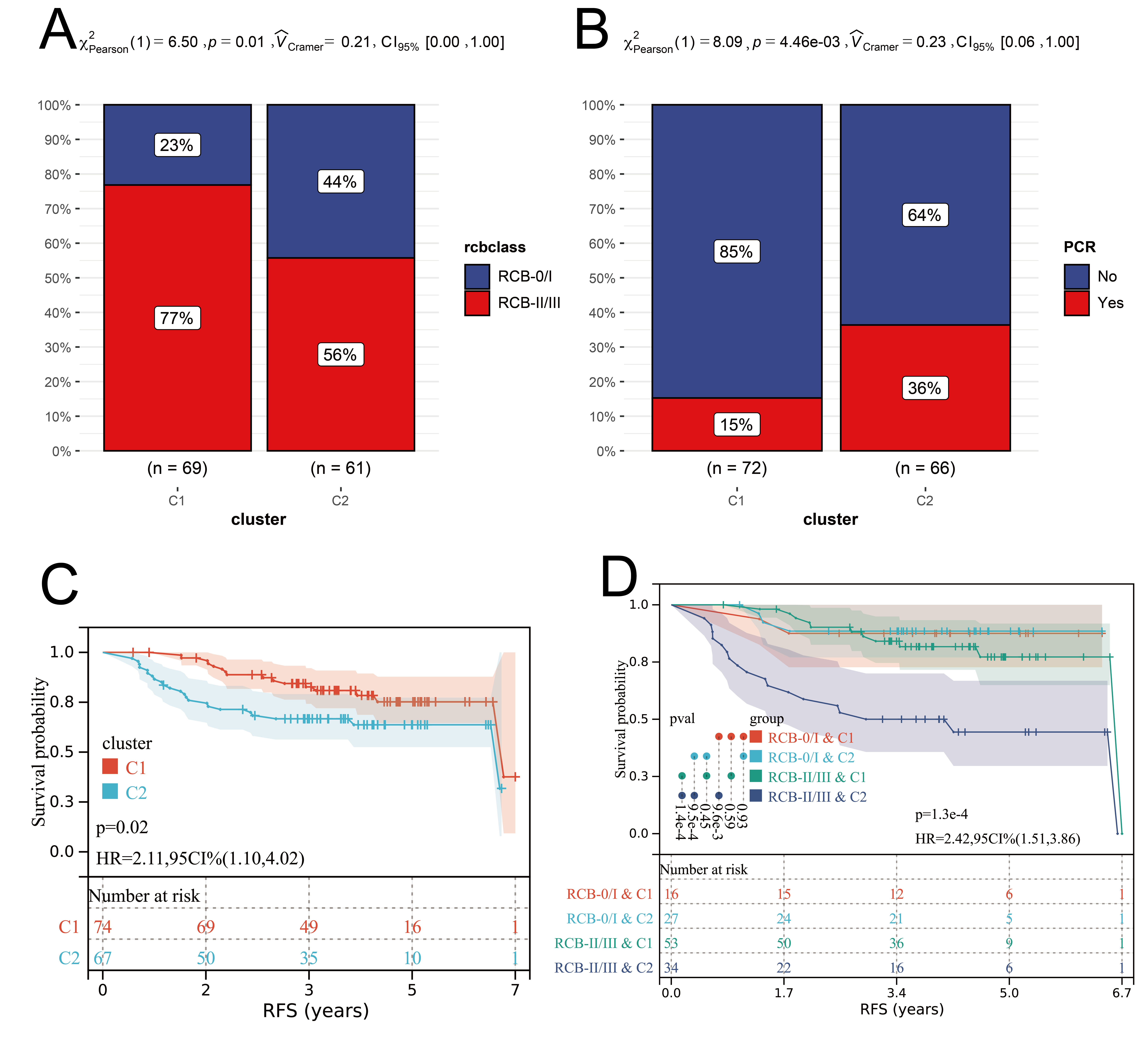

Supplement: Supplementary Figure 5 — Verification of the predictive ability of our molecular subtyping scheme in verification dataset (GSE32603). (A) The proportion of BC patients with different RCB class in Cluster C1 and Cluster C1 (Blue: RCB 0/I; Red: RCB II/III). (E) The proportion of BC patients achieved pCR after NAC in Cluster C1 and Cluster C1 (Blue: pCR-No; Red: pCR-Yes). (A) Differences in RFS between different molecular subtypes in GSE32603. (B) KM curve analysis of RFS is shown for patients classified according to molecular subtype and RCB class in GSE32603. [file Image_5.jpeg]

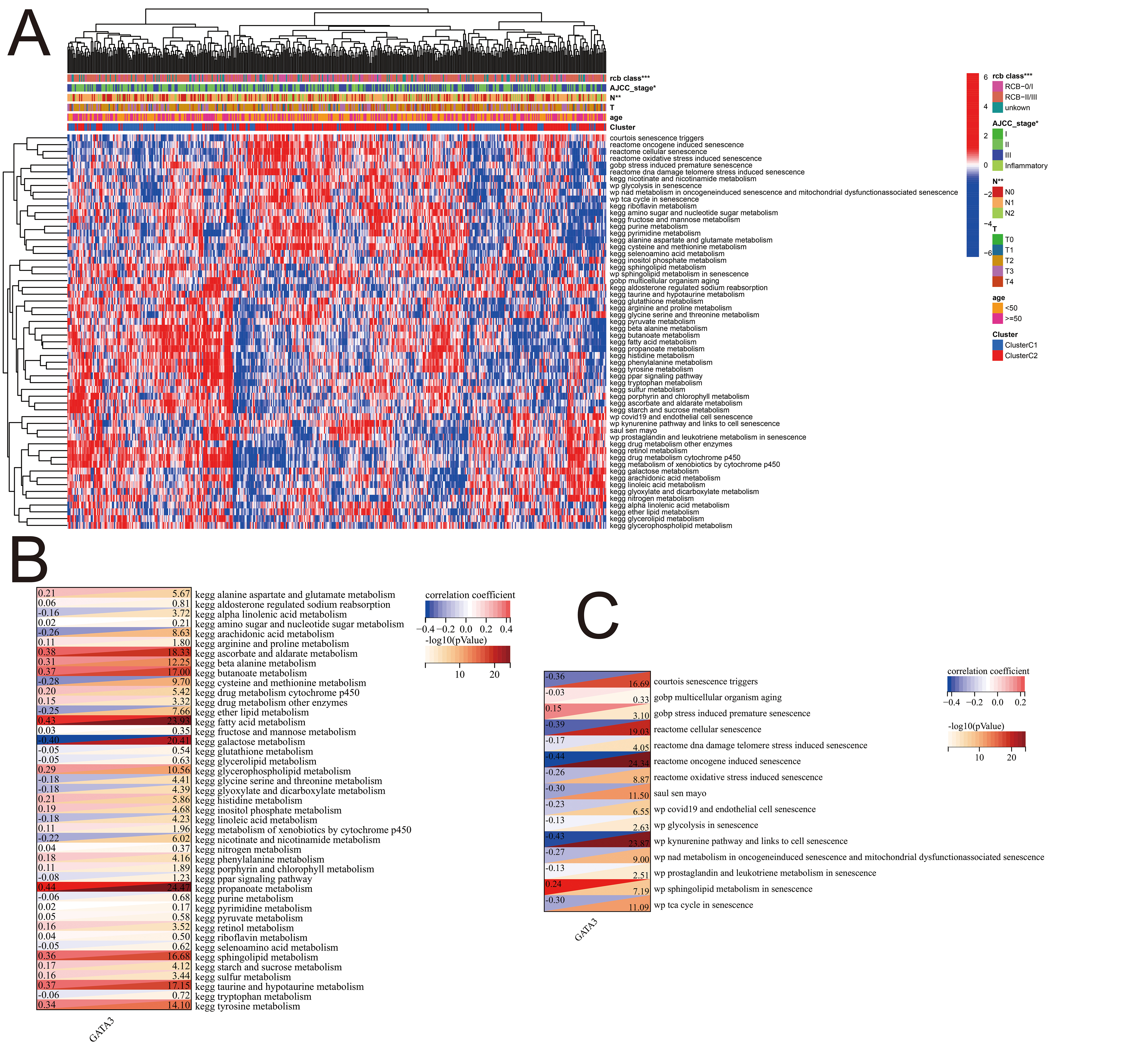

Supplement: Supplementary Figure 6 — (A) The heatmap displays the enrichment scores of the metabolism and senescence-related pathways between Cluster 1 and Cluster 2. The results of the Spearman correlation analysis between the expression level of GATA3 gene and the enrichment scores of the metabolism (B) and senescence (C) -related pathways. [file Image_6.jpeg]

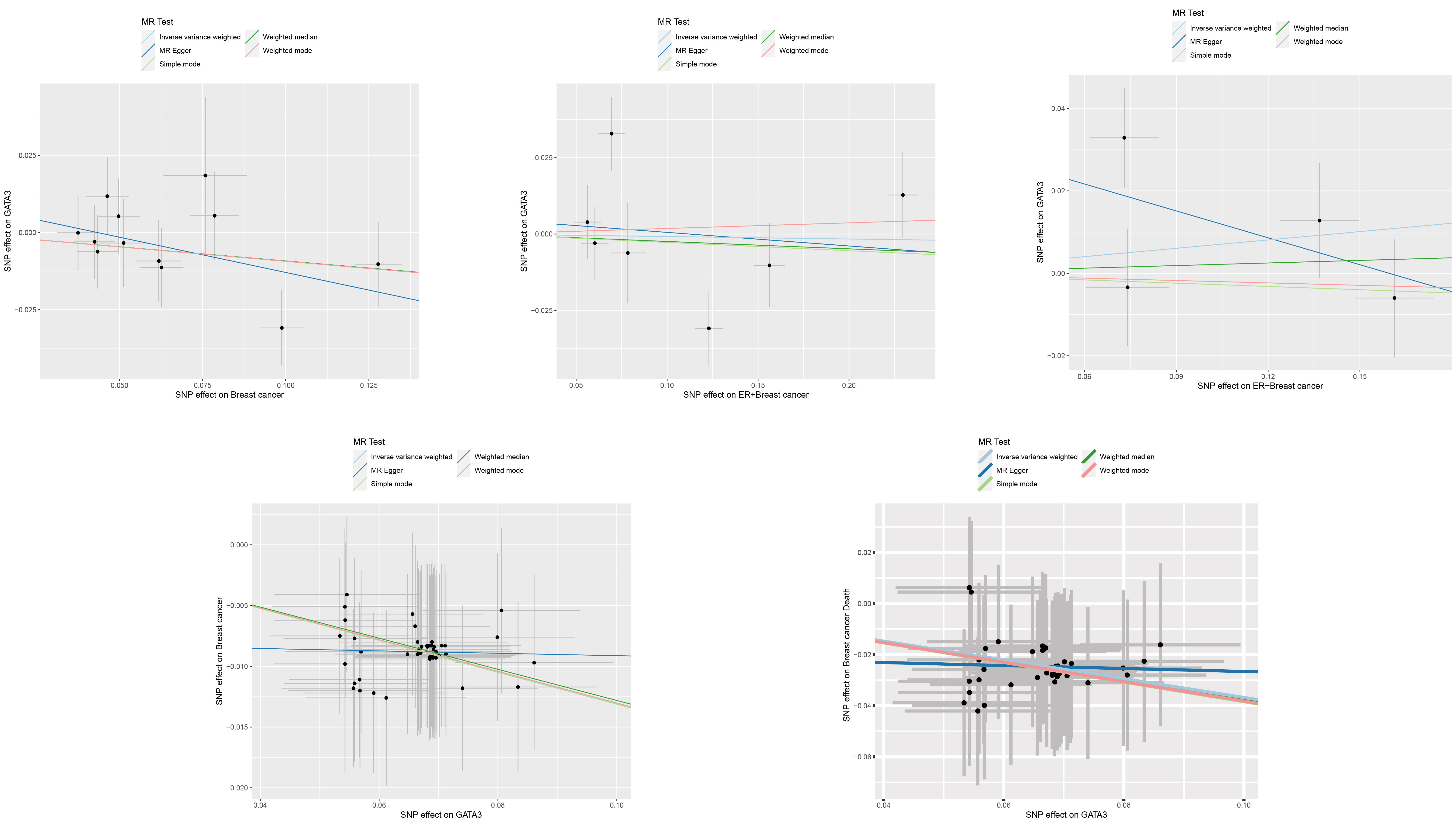

Supplement: Supplementary Figure 7 — The scatter plot of five MR methods. [file Image_7.jpeg]

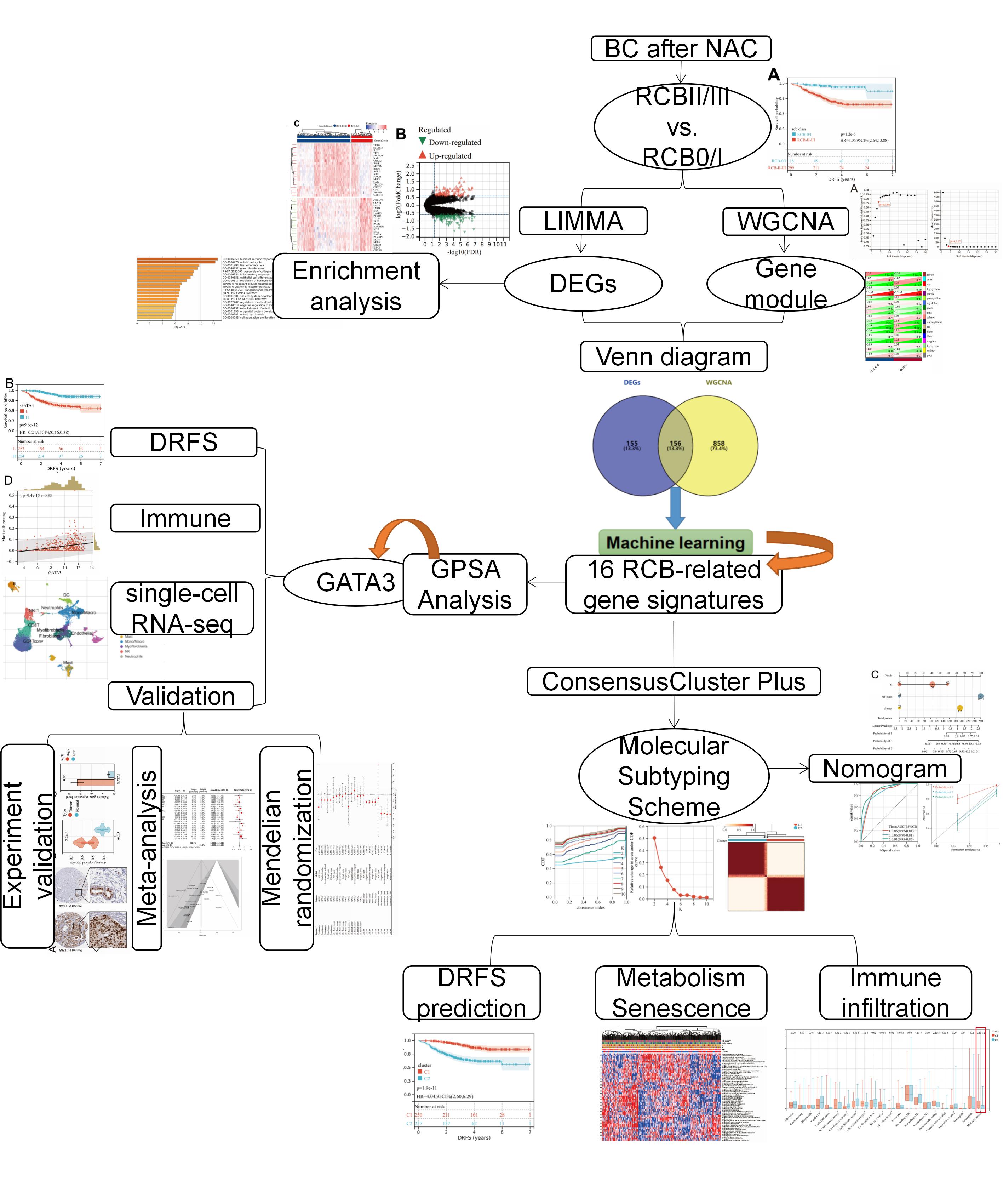

Supplement: Supplementary file 9 [file Image_8.jpeg]
